# Trans-cinnamaldehyde protects against phenylephrine-induced cardiomyocyte hypertrophy through the CaMKII/ERK pathway

**DOI:** 10.1186/s12906-022-03594-1

**Published:** 2022-04-25

**Authors:** Dongdong Qian, Jing Tian, Sining Wang, Xiaoli Shan, Pei Zhao, Huihua Chen, Ming Xu, Wei Guo, Chen Zhang, Rong Lu

**Affiliations:** 1 School of Basic Medical Science, Shanghai University of Traditional Chinese Medicine, Shanghai, 201203 China; 2 Department of Endocrinology, Shuguang Hospital Affiliated to Shanghai University of Traditional Chinese Medicine, Shanghai, 201203 China; 3 Department of Comprehensive Internal Medicine, Tongde Hospital of Zhejiang Province, Hangzhou, 310012 China; 4 Public Experiment Platform, School of Basic Medical Science, Shanghai University of Traditional Chinese Medicine, Shanghai, 201203 China; 5 Department of Physiology, Shanghai University of Traditional Chinese Medicine, Shanghai, 201203 China; 6 Department of Pathology, Shanghai University of Traditional Chinese Medicine, Shanghai, 201203 China

**Keywords:** Trans-cinnamaldehyde, Phenylephrine, Cardiac hypertrophy, CaMKII, ERK

## Abstract

**Background:**

Trans-cinnamaldehyde (TCA) is one of the main pharmaceutical ingredients of *Cinnamomum cassia Presl*, which has been shown to have therapeutic effects on a variety of cardiovascular diseases. This study was carried out to characterize and reveal the underlying mechanisms of the protective effects of TCA against cardiac hypertrophy.

**Methods:**

We used phenylephrine (PE) to induce cardiac hypertrophy and treated with TCA in vivo and in vitro. In neonatal rat cardiomyocytes (NRCMs), RNA sequencing and Kyoto Encyclopedia of Genes and Genomes (KEGG) pathway analysis were carried out to identify potential pathways of TCA. Then, the phosphorylation and nuclear localization of calcium/calmodulin-dependent protein kinase II (CaMKII) and extracellular signal-related kinase (ERK) were detected. In adult mouse cardiomyocytes (AMCMs), calcium transients, calcium sparks, sarcomere shortening and the phosphorylation of several key proteins for calcium handling were evaluated. For mouse in vivo experiments, cardiac hypertrophy was evaluated by assessing morphological changes, echocardiographic parameters, and the expression of hypertrophic genes and proteins.

**Results:**

TCA suppressed PE-induced cardiac hypertrophy and the phosphorylation and nuclear localization of CaMKII and ERK in NRCMs. Our data also demonstrate that TCA blocked the hyperphosphorylation of ryanodine receptor type 2 (RyR2) and phospholamban (PLN) and restored Ca^2+^ handling and sarcomere shortening in AMCMs. Moreover, our data revealed that TCA alleviated PE-induced cardiac hypertrophy in adult mice and downregulated the phosphorylation of CaMKII and ERK.

**Conclusion:**

TCA has a protective effect against PE-induced cardiac hypertrophy that may be associated with the inhibition of the CaMKII/ERK pathway.

**Supplementary Information:**

The online version contains supplementary material available at 10.1186/s12906-022-03594-1.

## Background

Myocardial hypertrophy is an adaptive response of the heart to a variety of pathological stimuli and is an independent risk factor for heart failure and sudden cardiac death [1]. In developing countries, death due to heart failure caused by cardiac hypertrophy accounts for approximately 25% of the total mortality [2, 3]. Preventing the occurrence and development of cardiac hypertrophy will be conducive to reducing the incidence and mortality of cardiovascular diseases.

The calcium/calmodulin-dependent protein kinase II (CaMKII) and mitogen-activated protein kinase (MAPK) pathways are two of the major cellular signalling pathways that drive cardiac hypertrophy. CaMKII is a multifunctional kinase that can phosphorylate target proteins containing serine or threonine residues [4]. CaMKII affects cardiac excitation-contraction coupling [5] and electrical activity [6] of normal cardiomyocytes by regulating some of the most important Ca^2+^ handling proteins, which promote the occurrence and development of myocardial hypertrophy and heart failure [7, 8]. CaMKII is also involved in the regulation of many transcription factors, which ultimately leads to cardiac hypertrophy and heart failure [9]. The mitogen-activated protein kinase (MAPK) signalling pathway plays a very important role in controlling a variety of physiological processes of cells, such as growth, development, division and death [10]. Many studies have reported that extracellular signal-related kinase (ERK1/2) signalling is involved in both adaptive and maladaptive cardiac hypertrophy [11].

Connections between CaMKII and ERK have been observed in many cell systems [12, 13]. In vascular smooth muscle cells (VSMCs) [14], the physical interaction of CaMKII and ERK regulates α1 adrenergic receptor (α1AR)-mediated proliferation. Using CaMKII selective inhibitors, Cipolletta E et al. demonstrated CaMKII-dependent ERK activation after PE stimulation and a reciprocal transactivation between CaMKII and ERK in cardiomyocytes [15]. Lu Y et al. found that transfection of cardiomyocytes with active CaMKII can cause activation of ERK and participate in activation of the foetal cardiac hypertrophy gene program; furthermore, they found that the CaMKII inhibitor KN93 can inhibit the activation of ERK1 induced by endothelin-1 [16].

*Cinnamomum cassia* is widely used in traditional Chinese medicine for the treatment of cardiovascular disease, kidney disease and joint disease [17]. Trans-cinnamaldehyde (TCA), a trans-isomer of cinnamaldehyde, is a kind of aromatic aldehyde extracted from cinnamon essential oils [18] and is the main active component of *Cinnamomum Cassia*. Studies have revealed that cinnamaldehyde has multiple pharmacological effects, including anticancer [19], anti-inflammatory [20, 21], sterilizing [22] and antioxidation [23, 24] effects. The role of cinnamaldehyde in cardiovascular diseases has also been investigated. In the process of diabetic cardiomyopathy, cinnamaldehyde can reduce the oxidative damage of cardiomyocytes by affecting the TRPA channel [25] and inhibit the production of inflammatory factors, inflammatory corpuscles and fibroblasts [26]. In a mouse model of acute myocardial injury induced by isoproterenol, cinnamaldehyde was found to reduce the expression of inflammatory factors in serum, the activity of SOD and the content of MAD, thus showing a cardioprotective role with increasing NO content [27]. In addition, studies have shown that cinnamaldehyde can affect the activity of L-type calcium channels (LTCCs) in cardiomyocytes [28, 29]. Moreover, cinnamaldehyde can reduce peripheral resistance and arterial blood pressure in dogs [30] and rats [31] via relaxation of large arteries and mesenteric arteries [32]. In a mouse model of myocardial hypertrophy induced by aortic banding surgery [33], cinnamaldehyde delayed the occurrence of myocardial hypertrophy and fibrosis by blocking the ERK signalling pathway. Similarly, Zhao H found that cinnamaldehyde can protect against LPS-induced cardiac dysfunction in rats by preventing MAPK pathway overactivation [34].

According to this evidence, in the present study, we investigated the potential underlying mechanism of TCA against PE-induced cardiomyocyte hypertrophy via transcriptome profile analysis and RNA-Seq. Then, based on the bioinformatics results, we further verified the therapeutic effects and underlying mechanisms of TCA in PE-induced cardiac hypertrophy in vivo and in vitro. Here, we observed that the CaMKII-ERK pathway was activated and that calcium homeostasis was disrupted by PE. We found that TCA can block the activation of the CaMKII and ERK signalling pathways and may therefore exert a protective effect against myocardial hypertrophy.

## Materials and methods

### Materials

Phenylephrine (PE) and trans-cinnamaldehyde (TCA) were purchased from Sigma–Aldrich (St. Louis, MO, USA). KN93 (CaM kinase II inhibitor), KN92 (Inactive analog of KN 93), 2-APB (IP3 receptor antagonist), and naftopidil hydrochloride (Naf, α1 antagonist) were obtained from TOCRIS (Bristol, UK). DMEM/F12, foetal bovine serum, penicillin and streptomycin were obtained from Gibco (Carlsbad, CA, USA). Fluo-4/AM and Pluronic™ F-127 (F-127) were purchased from Invitrogen (Carlsbad, CA, USA). Antibodies against MEK, ERK, p-MEK, and p-ERK as well as Alexa Fluor 488 phalloidin and Alexa Fluor 594 phalloidin antibodies were purchased from Cell Signaling Technology (Boston, MA, USA). Antibodies against CaMKII and p-CaMKII were obtained from Abcam (Cambridge, CB2 0AX, UK). Antibodies against GAPDH were purchased from Proteintech (Chicago, IL, USA).

### Neonatal rat cardiomyocyte (NRCM) culture and treatment protocol

NRCM cultures were prepared using a modified protocol, as previously described [35]. NRCMs were obtained from 1-day-old male Wistar rats, which were purchased from the Shanghai Laboratory Animal Center, Chinese Academy of Sciences (SLACCAS). The cells were cultured in a humidified incubator under 5% CO_2_ at 37 °C using Dulbecco’s Modified Eagle Medium/Nutrient Mixture F-12 [36, 37] containing 10% foetal bovine serum and 1% penicillin. Cells were treated with 50 μΜ phenylephrine for 24 h with the present or absence of different reagents, including 5 μΜ trans-cinnamaldehyde, 2 μΜ KN93, 1 μΜ KN92, 25 μΜ 2-APB, 10 μΜ Naf and 10 μΜ U0126.

### Transcriptome library construction and sequencing

Total RNA from the three groups (the control [CON], phenylephrine [PE], phenylephrine and trans-cinnamaldehyde [PE + TCA] groups) was extracted from cell samples using TRIzol reagent (Invitrogen, Carlsbad, CA, USA) following the manufacturer’s instructions. An mRNA-Seq library was constructed with a Hieff NGS™ MaxUp Dual-mode mRNA Library Prep Kit for Illumina® (YEASEN, 12301ES96, Shanghai, China) following the described procedures. Briefly, mRNAs were purified using oligo(dT)-attached magnetic beads and then fragmented and primed. The cleaved mRNA fragments were reverse-transcribed into first-strand cDNA, after which second-strand cDNA synthesis was performed. The double-stranded cDNAs were processed for end repair, poly(A) addition, and ligation with sequencing adaptors. Following purification, cDNA fragments 300 bp in size were selected and enriched by PCR amplification. The final libraries were subjected to paired-end sequencing on an Illumina HiSeq XTen platform (Shanghai Sangon Biotech Co., Ltd., Shanghai, China). The sequenced raw reads were filtered by removing adaptor sequences and evaluated by FastQC. The high-quality reads were assembled into transcripts after obtaining the accurate data with Trimmomatic. HISAT2 was used to compare the reads to the reference genome for mapping information. In the RNA-Seq analysis, gene expression levels were estimated by counting the reads located in genomic or exon regions. OmicsBean (http://www.omicsbean.com:39520/) was used to identify and analyse the differentially expressed genes (DEGs) through the DESeq2 process with a fold change > 1 and a *P* value < 0.05 as the criteria for significant differences between the two groups. The identified DEGs were then subjected to Gene Ontology (GO) functional analysis [38, 39] and Kyoto Encyclopedia of Genes and Genomes (KEGG) pathway analysis [40–42], and the results were visualized with an R package.

### Adult mouse cardiomyocyte (AMCM) isolation and culture

AMCMs were isolated and cultured as previously described [43]. Primary adult ventricular cardiomyocytes were isolated from 6- to 8-week-old C57BL/J mice. Under isoflurane anaesthesia, the heart was removed from the chest cavity and retrograde-perfused on a Langendorff apparatus with a collagenase solution (45 mg collagenase in 50 ml perfusion buffer consisting of 130 mM NaCl, 5 mM KCl, 0.5 mM NaH_2_PO_4_, 10 mM HEPES, 10 mM glucose, 10 mM BDM, 10 mM taurine, and 1 mM MgCl_2_). The softened cardiac tissue was aspirated with a pipette to form a cell suspension, which was then centrifuged at 300 rpm (rpm) for 5 min in Stop buffer (perfusion buffer with 5% sterile FBS). According to the different attachment velocities, the adult mouse cardiomyocytes were separated from other types of cells by centrifuging 3 times at 300 rpm for 5 min. In this process, the concentration of calcium was increased gradually to adapt to the calcium concentration in the culture medium. The culture medium included 50 ml of M199 (Gibco, Carlsbad, CA, USA), 0.1% albumin bovine V (Beyotime, Nanjing, China), 1% insulin-transferrin-sodium (Sigma, St. Louis, MO, USA), 0.5% CD lipid (Gibco, Carlsbad, CA, USA), 10 mM butanedione monoxime (BDM, Sigma, St. Louis, MO, USA) and 1% penicillin–streptomycin solution. Adult mouse cardiomyocytes cultured for 48 h were used to detect cardiomyocyte function and for calcium-related experiments.

### AMCM Ca^2+^ transients and sparks

Cells were incubated with 1 μmol/L Fluo-4/AM and F-127 for 20 min at 37 °C in the dark and then washed thrice with normal Tyrode’s solution. To measure calcium transients, the cells were stimulated with 1 Hz electric stimulation on a Carl Zeiss Axio Observer A1 microscope. The cells were illuminated with 488 nm light generated by a Lambda DG-4 monochromator and filter sets (Sutter, Novato, CA, USA) and recorded at 510 nm by using an EMCCD (iXon Ultra 897, Andor, Belfast, BT, UK).

### Measurement of sarcomere length

Cardiomyocyte SL was measured as described previously [44]. Cells were incubated with NT buffer for 3 min, and pictures were taken with a high-sensitivity digital CMOS camera (C11440-36 U, Hamamatsu Photonics K.K., Japan). Then, the contraction amplitude and other data were analysed offline with ImageJ and the SarcOptiM plugin.

### Mice and treatments

Cardiac hypertrophy was induced in vivo, as previously described [45]. Male 8-week-old C57BL/J mice weighing 19–20 g (purchased from Beijing Vital River Laboratory Animal Technology Co., Ltd.) were reared in the Experimental Animal Center of Shanghai University of Traditional Chinese Medicine in specific pathogen-free conditions with free access to food and a 12-h/12-h light/dark cycle. Briefly, PE (75 mg/kg/day) stimulation was maintained for 2 weeks through a subcutaneously implanted miniosmotic pump (Alzet, Model 2020; Durect, Cupertino, CA, USA). For pharmacological investigation, mice were treated via oral gavage daily with TCA at a dose of 50 mg/kg or 100 mg/kg or with normal saline.

### Echocardiography analysis

A high-resolution ultrasound imaging system (Vevo 2100, VisualSonics Inc., Toronto, Canada) was used to detect changes in cardiac structure and function. Under anaesthesia with 1% isoflurane in 95% oxygen and 5% carbon dioxide, the mice were fixed on the operating table in the supine position, and the heart rate was maintained at approximately 400 beats/min. M-mode recording was performed from the parasternal short-axis view, and LVPW, LVID, IVS, EF, and FS were measured.

### Histological analysis using haematoxylin and eosin (H&E) staining

Heart tissues were fixed in 4% paraformaldehyde for 48 h. They were dehydrated, embedded in paraffin, serially sectioned at 5 μm thickness, and processed by H&E staining. Images were acquired with an optical microscope (Carl Zeiss, Oberkochen, Germany).

### Western blot analysis

We extracted whole-cell protein from cells and tissues. Lysis buffer was added to the cells and tissues. After centrifugation, we obtained the protein supernatant. The protein supernatant was balanced, heated and denatured. The extracts from cardiomyocytes and myocardium were subjected to SDS–PAGE, and the proteins were then transferred to PVDF membranes. After soaking in 5% skimmed milk for 90 min, the membranes were probed overnight at 4 °C with primary antibodies against GAPDH (1:2000, Proteintech 10,021,642), P-CaMKII (1:1000, Abcam ab182647), CaMKII (1:1000, Abcam ab134041), P-MEK (1:1000, CST 9154S), MEK (1:1000, CST 4694S), P-ERK (1:1000, CST 9101S), and ERK (1:1000, CST 4696S). The membranes were rinsed with TBS containing 0.5% Tween 20 (TBST) three times and incubated with goat anti-mouse IgG-HRP (1:3000, Proteintech SA00001–1) and goat anti-rabbit IgG-HRP (1:3000, Proteintech SA00001–2) secondary antibodies for 1 h at room temperature. The blots were detected using an ECL system (Image Quant LAS 4000, GE Healthcare, Diegem, Belgium) and analysed with ImageJ software.

### Quantitative RT-PCR assay

To investigate the expression levels of hypertrophy-related genes, RNA was extracted from heart tissue using TRIzol (Invitrogen, Carlsbad, CA, USA), and its concentration was tested with a spectrophotometer (Thermo Fisher Scientific, Waltham, MA, USA). Reverse transcription was carried out with a Reverse Transcription Kit (Takara Bio, Kusatsu, Japan). Amplification was performed using SYBR Green Mastermix (Bio-Rad, Hercules, CA, USA) in 10 μl total reaction volumes on a Light Cycler® 480 Instrument II (Applied Biosystems, Bedford, MA, USA) with optimized thermocycling settings.

The following primer sequences were used: 5′-CTGGGACCCCTCCGATAGAT-3′ (forward) and 5′-TTCGGTACCGGAAGCTGTTG-3′ (reverse) for Nppa, 5′-GGAGAACACGGCATCATTGC-3′ (forward) and 5′-CTCCAGCAGCTTCTGCATCT-3′ (reverse) for Nppb, 5′-ATGTGCCGGACCTTGGAAG-3′ (forward) and 5′-CCTCGGGTTAGCTGAGAGATCA-3′ (reverse) for Myh7, and 5′-GTAACCCGTTGAACCCCATT-3′ (forward) and 5′-CCATCCAATCGG-TAGTAGCG-3′ (reverse) for 18S rRNA.

### Immunofluorescence staining

NRCMs were seeded on glass plates, fixed with 4% paraformaldehyde and rinsed with PBS three times. Then, these NRCMs were permeabilized with 0.5% Triton X-100 for 5 min and blocked with 10% BSA for 1 h at room temperature. The samples were incubated overnight at 4 °C with primary antibodies against α-actinin (1:100, Abcam ab9465), p-ERK (1:100, CST 9101S), p-CaMKII (1:100, Abcam ab182647), RyR2 (1:100, Abcam ab196355), and PLN (1:100, Santa sc-21,923). The plates were incubated with anti-rabbit IgG (H + L) Alexa 488 (1:200, CST 4412 s, for p-ERK, p-CaMKII, p-RyR2 and p-PLN probing) and anti-mouse IgG (H + L) Alexa 594 (1:200 CST 8890 s, for actinin probing) secondary antibodies in the dark for 1 h at room temperature. The cells were observed and photographed under a Zeiss LSM880 confocal microscope (Carl Zeiss, Jena, Germany).

### Statistical analysis

All results are expressed as the mean ± SEM. The data were analysed with one-way ANOVA, and group differences were detected using a Newman–Keuls test when the initial ANOVA revealed significant differences. *P* < 0.05 was considered to indicate significance.

## Results

### TCA altered various signalling pathways in myocytes in PE-induced hypertrophy myocytes

**Fig. 1:**
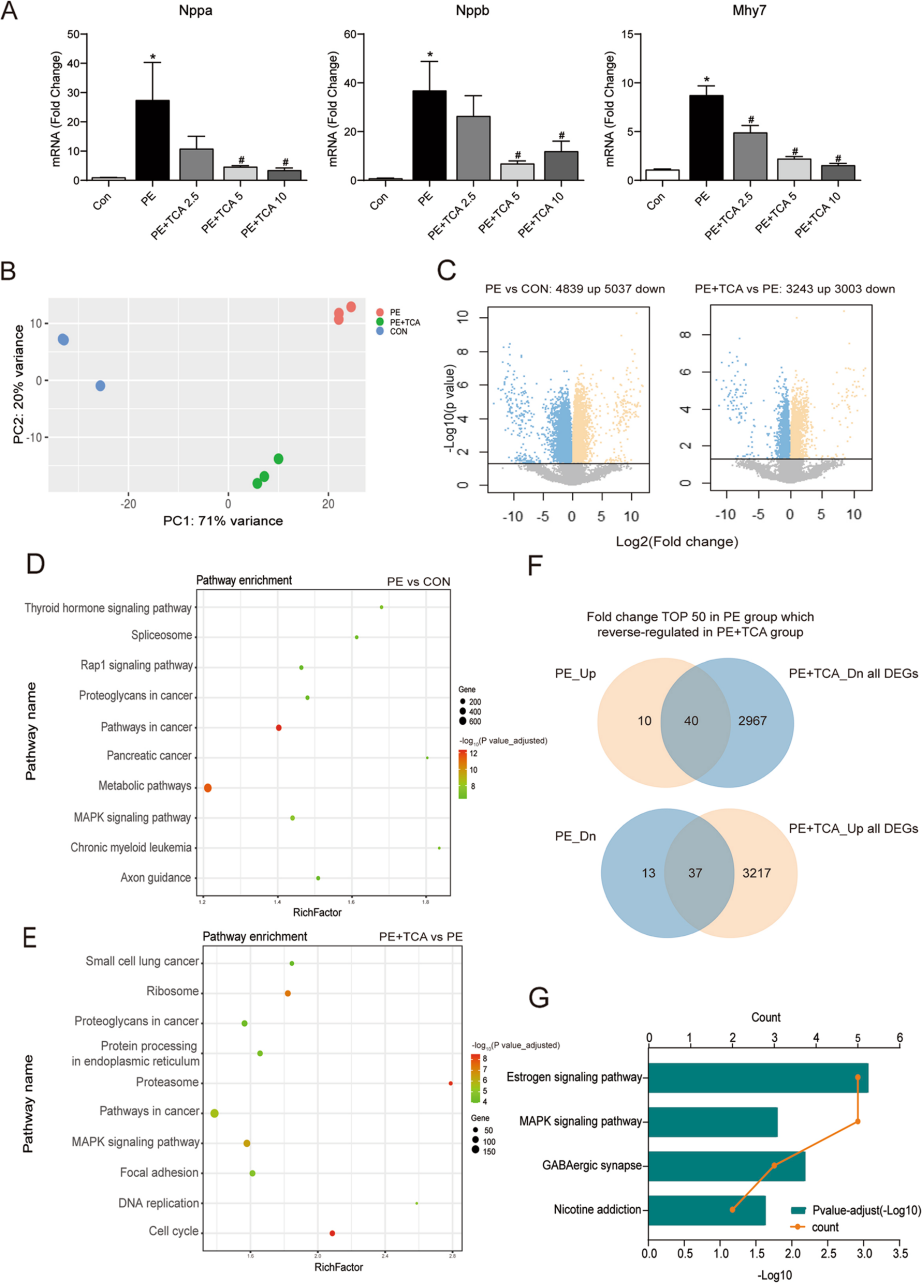
TCA altered various signalling pathways in myocytes in PE-induced hypertrophy myocytes. **A** A dose-response relationship between hypertrophic genes and TCA- treated with different concentrations in 2.5 μM, 5 μM and 10 μM. **B** Principal component analysis of the control group (CON), the phenylephrine group (PE), the phenylephrine and trans-cinnamaldehyde group (PE + TCA). **C** Differential genes volcano plots in the PE group vs. the control group and the PE + TCA group vs. the PE group. **D-E** KEGG pathway enrichment of differential genes in the PE group vs. the control group and the PE + TCA group vs. the PE group. **F** Venn diagram showing the overlapped genes between fold change TOP 50 in the PE group which reverse-regulated in the PE + TCA group. **G** KEGG enrichment of differential genes. These data are expressed as the mean ± SEM. *P < 0.05 versus control; #*P* < 0.05 versus PE. PE: phenylephrine; TCA: trans-cinnamaldehyde

To clarify the potential protective mechanism of TCA against PE-induced cardiac hypertrophy, we performed RNA-Seq on NRCMs. However, first, a dose–response relationship between TCA and hypertrophic genes was observed at different concentrations (2.5 μM, 5 μM, and 10 μM) (Fig. 1A). The concentration of TCA in the following experiments was 5 μM. Then, we extracted RNA from cells (including cells in the control group, PE group and PE + TCA group) and sequenced it with high-throughput technology to observe the differences in gene expression and perform transcriptome analysis. Through principal component analysis, the gene expression of the groups was found to be disparate (Fig. 1B). There were 4839 upregulated genes and 5037 downregulated genes in PE-treated cells compared with control cells. In addition, 3243 genes were upregulated and 3003 genes were downregulated in the TCA group compared with the PE group (Fig. 1C). The KEGG pathway enrichment analysis revealed 162 signalling pathways for the PE group vs. the control group, of which the first 10 pathways with the highest adjusted *P* values are shown in Fig. 1D. Similarly, KEGG enrichment analysis revealed 116 pathways in the TCA group vs. the PE group, of which the first 10 pathways are shown in Fig. 1E. Then, the top 50 upregulated genes and top 50 downregulated genes upon PE treatment were analysed with a Venn diagram. As shown in Fig. 1F, 77 genes were reverse-regulated by TCA. These genes were also subjected to KEGG enrichment analysis, which found that there were 4 pathways (Fig. 1G). The MAPK signalling pathway was the only one in all 3 KEGG pathway analyses, which indicates that it may be involved in the therapeutic effects of TCA against PE-induced cardiac hypertrophy.

### The CaMKII-MEK-ERK signalling pathway was involved in PE-induced cardiac hypertrophy

**Fig. 2:**
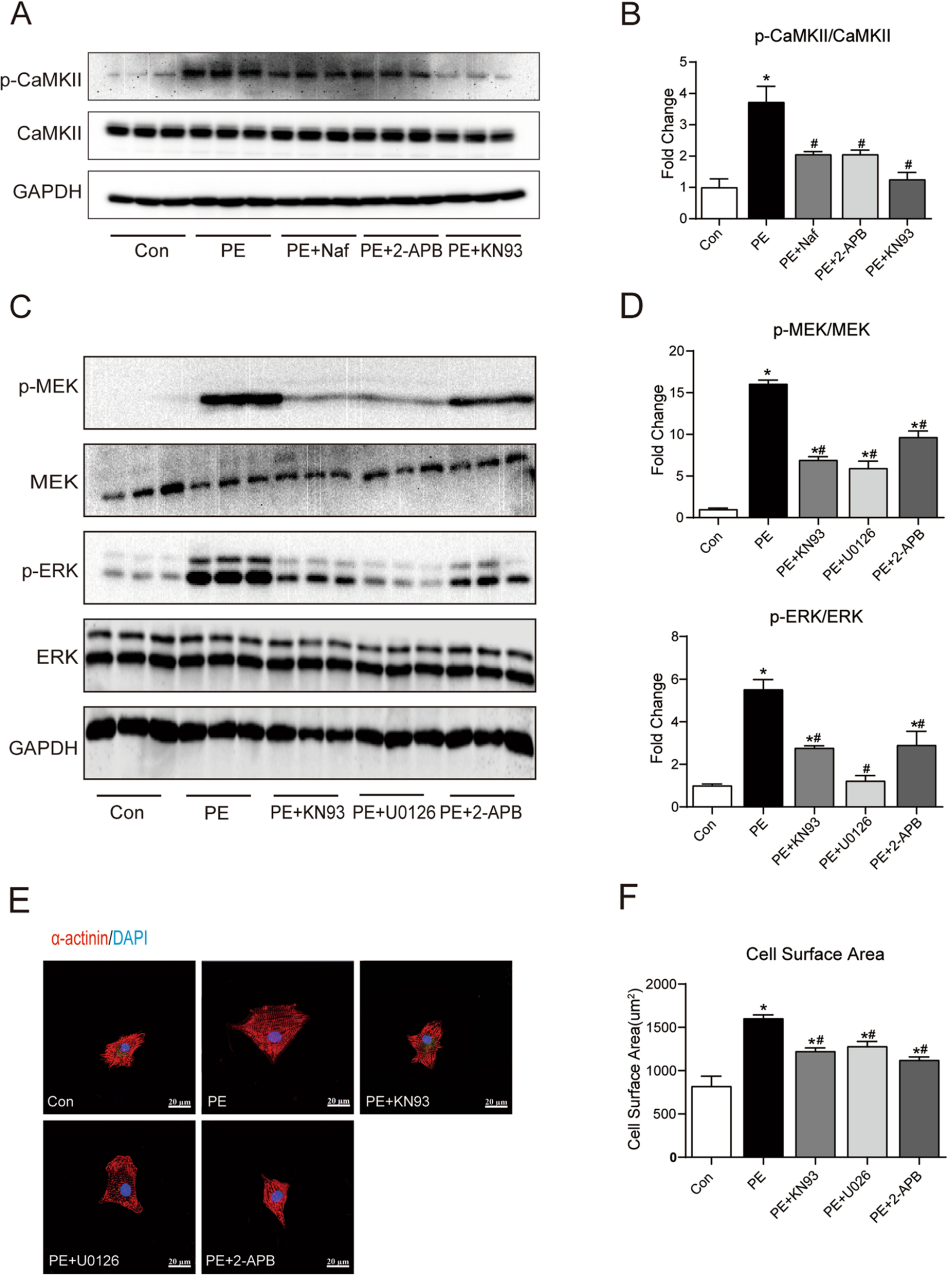
The CaMKII-MEK-ERK signalling pathway was involved in PE-induced cardiac hypertrophy. **A** Representative immunoblots of CaMKII and GAPDH. **B** Quantification analysis of CaMKII. **C** Representative immunoblots of p-MEK, MEK, p-ERK, ERK and GAPDH. **D** Quantification analysis of MEK and ERK. **E** Representative confocal fluorescent images of α-actinin. Red: α-actinin; Blue: DAPI. **F** Analysis of cell surface area according to α-actinin immunofluorescence staining. *P < 0.05 versus control; #*P* < 0.05 versus PE. Naf: an α1-adrenergic receptor antagonist; 2-APB: an IP3 receptor antagonist; KN93: a CaMKII inhibitor; U0126: a MEK/ERK inhibitor

The CaMKII-ERK pathway has been proposed to promote PE-induced cardiac hypertrophy [15, 16]. However, there is limited understanding of the mechanism. Accordingly, we verified that this signalling pathway plays a role in PE-induced cardiac hypertrophy in NRCMs. As shown in Fig. 2A and B, our data consistently demonstrated that PE treatment enhanced the phosphorylation level of CaMKII, an effect that was relieved by Naf (an α1-adrenergic receptor antagonist), 2-APB (an IP3 receptor antagonist), and KN93 (a CaMKII inhibitor). PE treatment consistently increased the phosphorylation levels of MEK and ERK1/2, but these increases were largely blocked by KN93, U0126 (a MEK/ERK inhibitor) and 2-APB (Fig. 2C and D). Moreover, cardiac hypertrophy was assessed by measuring the cellular surface area. As shown in Fig. 2E and F, PE treatment increased the cellular surface area, but this effect was largely blocked by 2-AB, KN93, and U0126. These data suggest that the CaMKII-MEK-ERK pathway plays a role in PE-induced cardiac hypertrophy.

### TCA ameliorated PE-induced cardiomyocyte hypertrophy through the CaMKII-MEK-ERK pathway

**Fig. 3:**
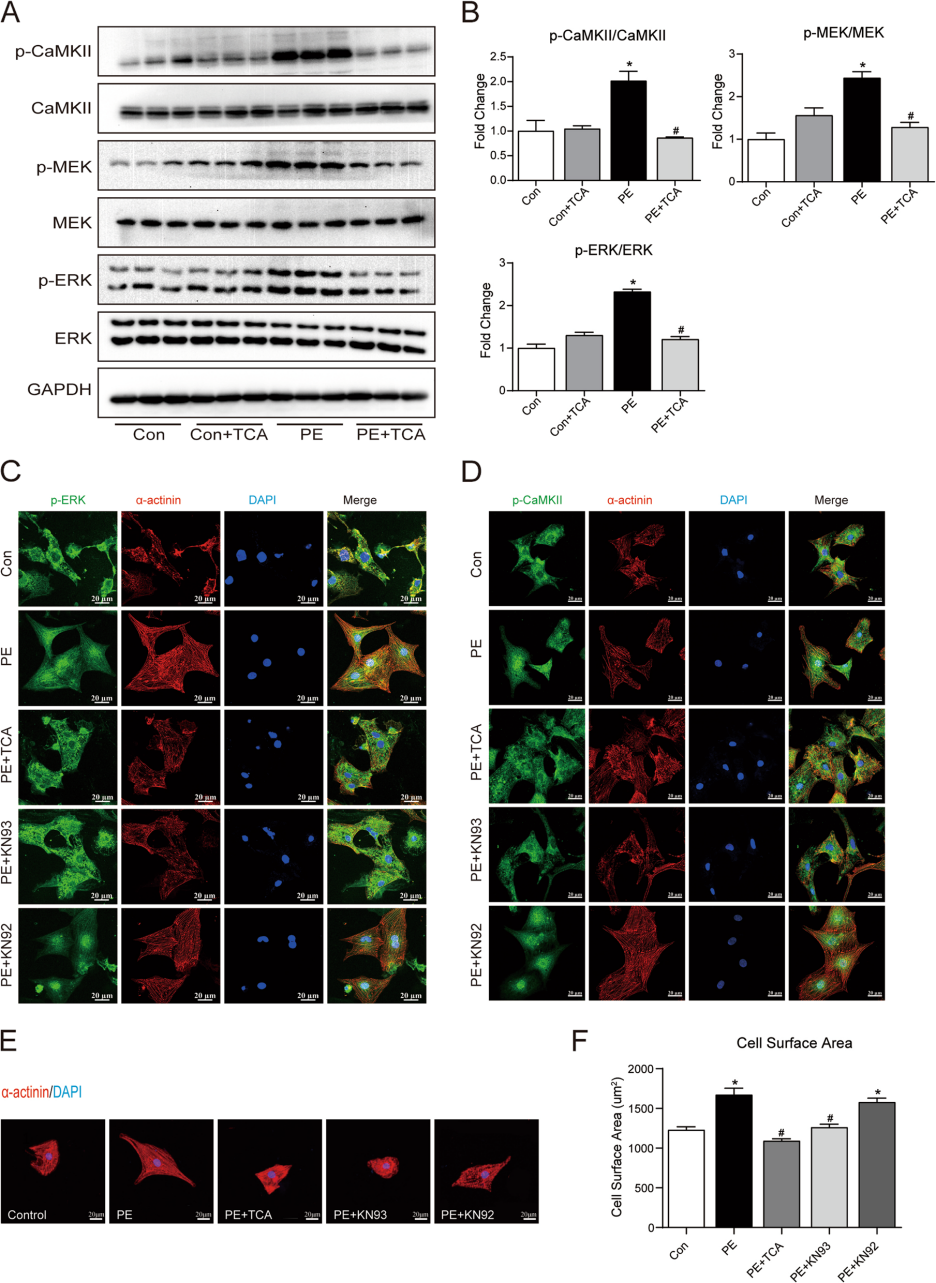
TCA ameliorated PE-induced cardiomyocyte hypertrophy through the CaMKII-MEK-ERK pathway. **A**-**B** Representative blots and quantification of p-CaMKII/CaMKII, p-MEK/MEK and p-ERK/ERK in NRCMs. **C**-**D** Representative immunofluorescence images showing p-CaMKII and p-RyR2. **E** Representative confocal fluorescent images of α-actinin. Red: α-actinin; Blue: DAPI. **F** Analysis of cell surface area according to α-actinin immunofluorescence staining. *P < 0.05 versus control; #*P* < 0.05 versus PE. KN92: Non activated derivatives of KN93

Then, we investigated whether TCA protects against PE-induced cardiac hypertrophy through the MAPK signalling pathway. Accordingly, western blot analysis was carried out, and the results showed that the phosphorylation levels of CaMKII, MEK, and ERK were significantly increased after PE stimulation, while TCA reversed this change (Fig. 3A and B). TCA alone did not significantly change the phosphorylation level of CaMKII, MEK, or ERK. In a resting state, CaMKII and ERK1/2 reside in the cytoplasm. Immunofluorescence assays demonstrated that PE stimulation caused dramatic nuclear localization of p-CaMKII and p-ERK that was largely blocked by TCA and KN93 but not by KN92, an inactive analogue of KN93 (Fig. 3C and D). Next, we observed myocyte hypertrophy by assessing surface area. As shown in Fig. 3E-F, PE treatment increased the surface area of cardiomyocytes. This effect was ameliorated by TCA or KN93 but not by KN92. These data suggest that TCA inhibits PE-induced cardiomyocyte hypertrophy, protein phosphorylation and nuclear translocation through the CaMKII pathway.

### TCA restored calcium handling and sarcomere contractility

**Fig. 4:**
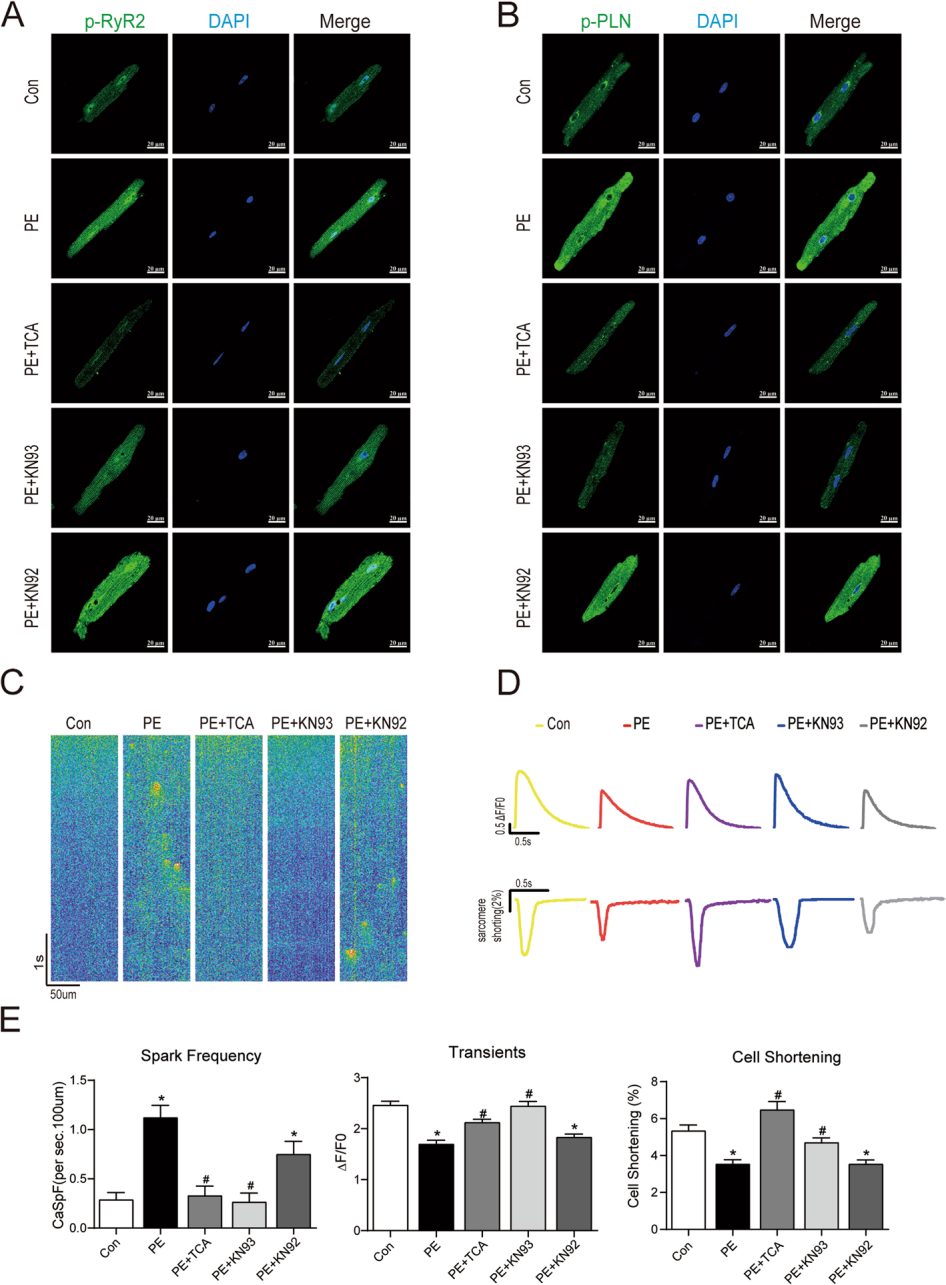
TCA restored calcium handling and sarcomere contractility. **A**-**B** Representative immunofluorescence images of p-PLN and p-RyR2. Green: p-PLN and p-RyR2; Blue: DAPI; **C** Diagrammatic sketch of the calcium sparks. **D** Average traces of Ca^2+^ transients (above) and cell shortening (down) from Fluo-4/AM and F-127 loaded cardiomyocytes isolated from the heart of adult mice. **E** Quantification of Ca^2+^ spark frequency, Ca^2+^ transients and cell shortening. *P < 0.05 versus control, #*P* < 0.05 versus PE. PLN: phospholamban; RyR2: ryanodine receptor type 2

CaMKII is one of the key players in myocyte excitation-contraction coupling [5]. We investigated whether TCA can also restore myocyte excitation-contraction coupling. Given that Ca^2+^ signals in neonatal rat ventricular myocytes are different from those in adult myocytes [46], we examined how TCA affects excitation-contraction coupling in adult rat ventricular myocytes. Immunofluorescence assays demonstrated that PE robustly increased the phosphorylation of PLN and RyR2 and that this effect was reversed by TCA and KN93 but not by KN92 (Fig. 4A and B). To reveal how TCA affects Ca^2+^ handling, we measured SR Ca^2+^ spark frequency, Ca^2+^ transients and sarcomere shortening. Our data showed that PE increased the Ca^2+^ spark frequency, an effect that was largely blocked by TCA and KN93 but not by KN92 (Fig. 4C and E). Our data also demonstrated that Ca^2+^ transient amplitudes and sarcomere shortening were depressed by TCA. This depression was largely prevented by TCA and KN93 but not by KN92 (Fig. 4D and E).

### TCA abrogated PE-induced cardiac hypertrophy in vivo

**Fig. 5:**
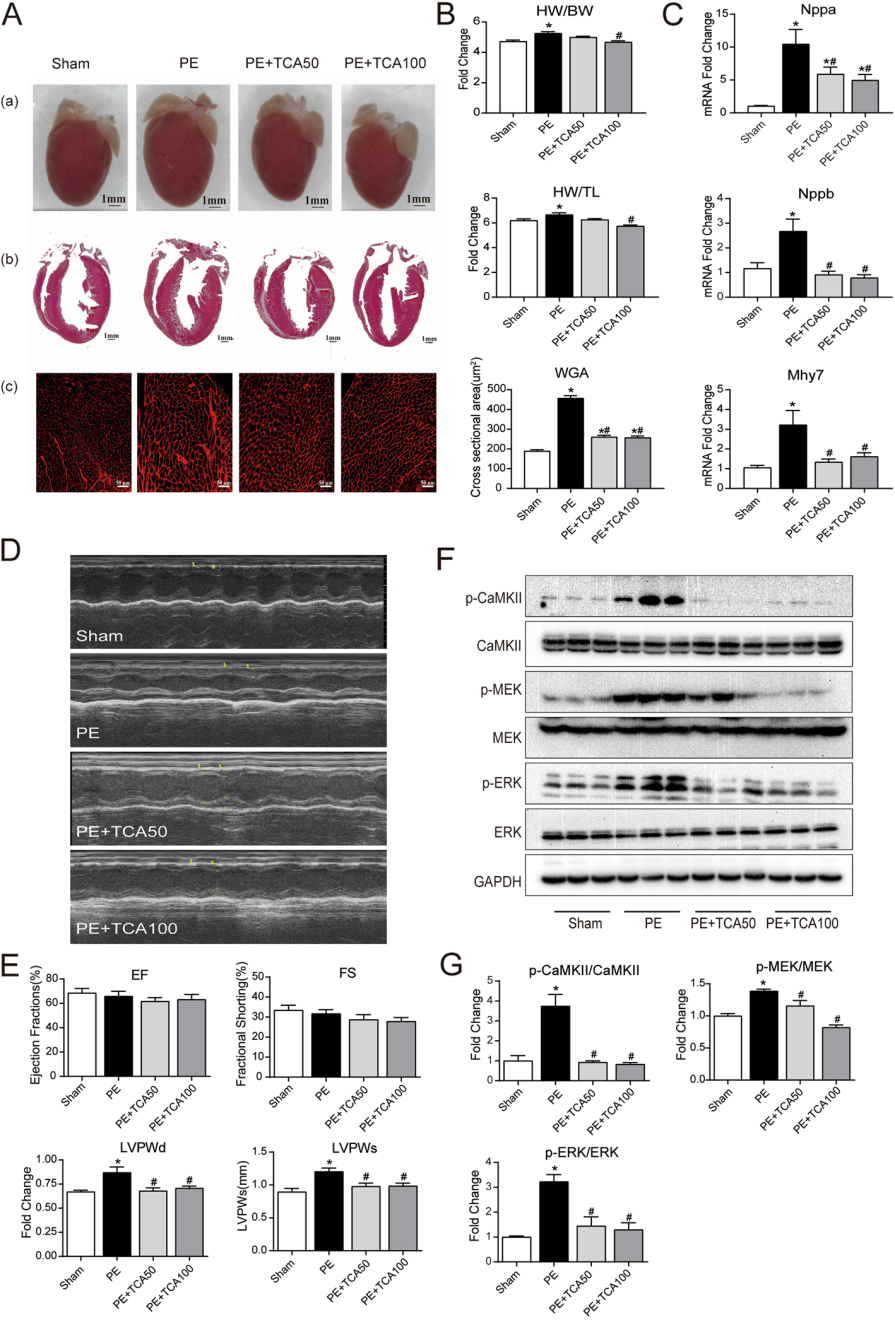
TCA abrogated PE-induced cardiac hypertrophy in vivo*.***A** Representative myocardium pictures. (a) Pictures of heart material object; (b) H&E staining heart sections from paraffin embedding samples; (c) WGA staining from heart frozen sections. **B** Quantification of heart weight/body weight ratio, heart weight/tibial length ratio and cross-section with WGA staining. **C** mRNA expression of Nppa, Nppb and Mhy7 in myocardium tissues. **D** Diagram of echocardiography. **E** Quantification of EF, FS, LVPWd and LVPWs. **F** Immunoblotting in detection of p-CaMKII, CaMKII, p-MEK, MEK, p-ERK and ERK in myocardial tissue. **G** Quantification of p-CaMKII/CaMKII, p-MEK/MEK, p-ERK/ERK. *P < 0.05 versus control; #*P* < 0.05 versus PE. EF: ejection fraction; FS: fractional shorting

We next attempted to determine whether the antihypertrophic effects of TCA in cultured myocytes could also be seen in vivo in mice subjected to 2 weeks of subcutaneous administration of PE. Morphological observation and WGA staining assays suggested that TCA might prevent PE-induced cardiac hypertrophy (Fig. 5A). These findings were consistent with the gravimetric measurements. As shown in Fig. 5B, TCA at a dose of 100 mg/kg/d, but not 50 mg/kg/d, blocked the increases in the heart weight-to-body weight ratio and heart weight-to-tibia ratio. Several hypertrophic genes, including Nppa, Nppb and Mhy7, were upregulated in the PE group, but this upregulation was prevented by TCA administration (Fig. 5C). Ultrasound imaging revealed that the interventricular septal thickness at both systole and diastole increased with PE stimulation, but this effect was prevented by TCA. The ultrasound results also demonstrated that there were no significant differences in ejection fraction (EF) or fractional shortening (FS) among those groups (Fig. 5D and E). We then investigated whether TCA could protect against PE-induced hyperactivity of the CaMKII-ERK pathway in vivo. As shown in Fig. 5F and G, after PE stimulation, the phosphorylation of CaMKII, MEK and ERK increased, and this effect was largely blocked by TCA at both doses. These data confirm that TCA inhibits the PE-induced hyperphosphorylation of CaMKII-MEK-ERK and ameliorates PE-induced cardiac hypertrophy in vivo.

## Discussion

In the present study, we conducted a transcriptomic analysis of the protective effects of TAC against cardiac hypertrophy in myocytes. In KEGG pathway enrichment analysis, the MAPK signalling pathway was among the most enriched pathways. These data suggest that the MAPK signalling pathway may be related to the therapeutic effects of TCA.

Studies have revealed that PE, an α1-adrenergic receptor agonist, can activate the CaMKII signalling pathway, which ultimately leads to cardiac hypertrophy [47]. In the heart, CaMKII has been considered to be the key influencer of excitation-contraction coupling (ECC) [48] and excitation-transcription coupling (ETC) [49]. In the physiological state, CaMKII activity undergoes self-regulation according to the intracellular calcium content and is activated by posttranslational modifications [50–52]. Increasing the expression of CaMKII leads to hypertrophic gene expression, ultimately causing myocardial hypertrophy and heart failure [9]. Previous studies have also shown that the ERK pathway is involved in hypertrophy induced by phenylephrine [53]. Upon activation by pathological factors, ERK1/2 translocates to the nucleus, where it phosphorylates transcription factors, such as NF-κB, Nrf2, and MEF2 [54–56], and in turn induces the mRNA expression of hypertrophy-related genes, such as atrial natriuretic peptide (ANP) [57].

A previous study has demonstrated that CaMKII-ERK crosstalk promotes cardiac hypertrophy and that targeting the CaMKII-ERK pathway is effective in reducing cardiac hypertrophy [15]. This is consistent with our data, especially the finding that KN93 blocked PE-induced cardiac hypertrophy and hyperphosphorylation of the MEK-ERK pathway. Our data also demonstrated that PE-induced hyperphosphorylation of CaMKII, MEK and ERK1/2 and PE-induced nuclear localization of CaMKII and ERK were largely prevented by TCA administration. Based on our current data, it is unclear whether TCA is directly suppressing MEK and ERK1/2 or by suppressing the upstream signaling pathway. But considering that TCA alone slightly enhanced phosphorylation level of MEK and ERK, TCA more likely to block PE-induced hyperphosphorylation of MEK and ERK via a CaMKII-MEK-ERK pathway.

CaMKII is involved in the regulation of cardiac myocyte Ca^2+^ handling through phosphorylation of several key players, including phospholamban (PLN) and ryanodine receptor type 2 (RyR2 [58, 59]. In the failing heart, hyperactivity of CaMKII directly phosphorylates RyR2, mostly at the Ser2814 site [60, 61], following the opening of the RyR2 channel, leading to an increase in calcium leakage. Excessive calcium leakage can lead to insufficient calcium content within the sarcoplasmic reticulum and affects the contraction of cardiomyocytes. Our data showed that TCA largely prevented PE-induced hyperphosphorylation of PLN and RyR2 as well as Ca^2+^ mishandling. According to our data in Fig. 5E, it shows that FS in TCA groups seems depressed, though there are no significance differences among those groups. Despite of this, a long term or higher dosage of TCA treatment should be carried in future studies to clarify if TCA will supress FS.

## Conclusions

**Fig. 6:**
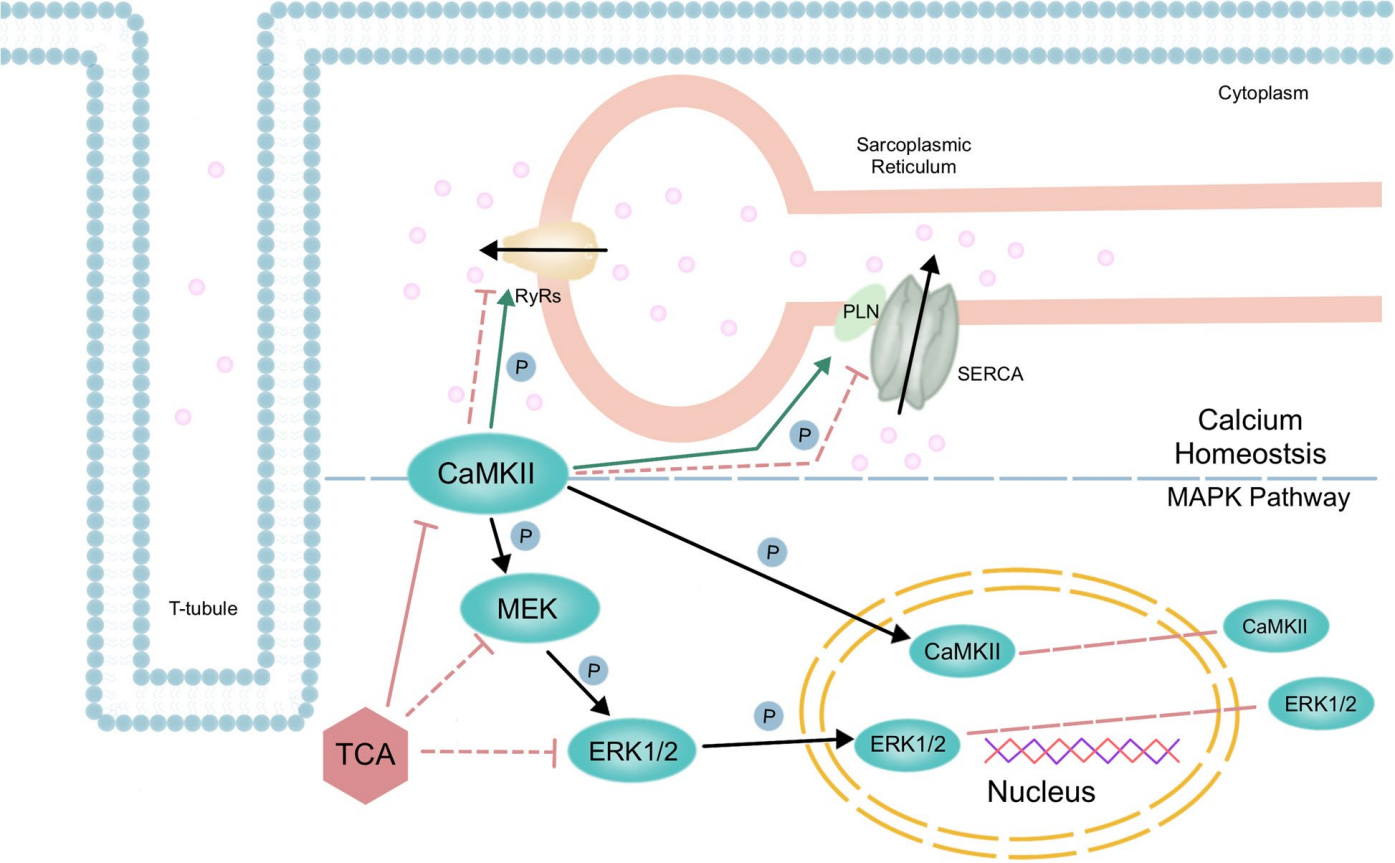
Protective effects and possible mechanism of TCA on PE-induced cardiac hypertrophy. TCA protects PE-induced cardiac hypertrophy via blocking CaMKII-ERK activation and nuclear localization. TCA prevents PE-induced CaMKII activation which also may contribute to restoring cardiac excitation-contraction coupling. CaMKII: calcium/calmodulin-dependent protein kinase II; ERK: extracellular regulated protein kinases; MAPK: mitogen activated protein kinase; PE: phenylephrine; PLN: phospholamban; RyR2: ryanodine receptor type 2; SERCA: sarcoplasmic/endoplasmic reticulum Ca^2+^-ATPase; TCA: trans-cinnamaldehyde

Collectively, our findings demonstrate that TCA prevents PE-induced hyperphosphorylation and nuclear localization of CaMKII and ERK and restores myocyte calcium homeostasis (Fig. 6). Based on these results, we hypothesize that prevention of hyperphosphorylation of the CaMKII-ERK pathway plays a role in the therapeutic effects of TCA against cardiac hypertrophy.

## Supplementary Information

**Additional file 1: Figure S1**. Chemical structure of trans-cinnamaldehyde (TCA). **Figure S2-S5**. Original blot images.

## Data Availability

The datasets used and/or analyzed during the current study available from the corresponding author on reasonable request.

## Abbreviations

BW: Body weight
CaMKII: Calcium/calmodulin-dependent protein kinase II
ECC: Excitation contraction coupling
EF: Ejection fraction
ER/SR: Endo (sarco) plasmic reticulum
ERK: Extracellular regulated protein kinases
FS: Fractional shorting
HE: Hematoxylin-eosin staining
HW: Heart weight
FS: Fractional shortening
LVPWd: Diastolic left ventricular posterior wall thickness
LVPWs: Systolic left ventricular posterior wall thickness
LTCC: L-type calcium channel
MAPK: Mitogen activated protein kinase
MHC-β: myosin heavy chain beta
Nppa: Atrial natriuretic peptide (ANP)
Nppb: Brain natriuretic peptide (BNP)
NRCM: Neonatal rat cardiac myocyte
PE: Phenylephrine
PLN: Phospholamban
RyR2: Ryanodine receptor type 2
SERCA: Sarcoplasmic/endoplasmic reticulum Ca^2+^-ATPase
TCA: Trans-cinnamaldehyde
TL: Tibial length
WGA: Wheat germ agglutinin
