# Supplementary material for: Trans-cinnamaldehyde protects against phenylephrine-induced cardiomyocyte hypertrophy through the CaMKII/ERK pathway
Source: BMC Complement Med Ther. 2022 Apr 25;22:115. doi: 10.1186/s12906-022-03594-1 (PMC9040265; doi:10.1186/s12906-022-03594-1)
Supplement: Supplementary file 1 — Additional file 1: Figure S1. Chemical structure of trans-cinnamaldehyde (TCA). Figure S2-S5. Original blot images. [file 12906_2022_3594_MOESM1_ESM.pdf]

Supplementary Information

Trans-cinnamaldehyde protects against Phenylephrine-induced Cardiomyocyte Hypertrophy Through CaMKII/ERK Pathway

Dongdong Qian[1†], Jing Tian[1,2†], Sining Wang[3], Xiaoli Shan[4], Pei Zhao[4], Huihua Chen[1], Ming Xu[5], Wei Guo[6], Chen Zhang[6*] and Rong Lu[1*]

[1] School of Basic Medical Science, Shanghai University of Traditional Chinese Medicine, Shanghai 201203, China.

[2] Department of Endocrinology, Shuguang Hospital Affiliated to Shanghai University of Traditional Chinese Medicine, Shanghai 201203, China.

[3] Department of Comprehensive Internal Medicine, Tongde Hospital of Zhejiang Province, Hangzhou 310012, China.

[4]Public Experiment Platform, School of Basic Medical Science, Shanghai University of Traditional Chinese Medicine, Shanghai 201203, China.

[5] Department of Physiology, Shanghai University of Traditional Chinese Medicine, Shanghai 201203, China.

[6] Department of Pathology, Shanghai University of Traditional Chinese Medicine, Shanghai 201203, China.

*** Corresponding author：**

Chen Zhang: zhangchen@shutcm.edu.cn;

Rong Lu: lurong@shutcm.edu.cn

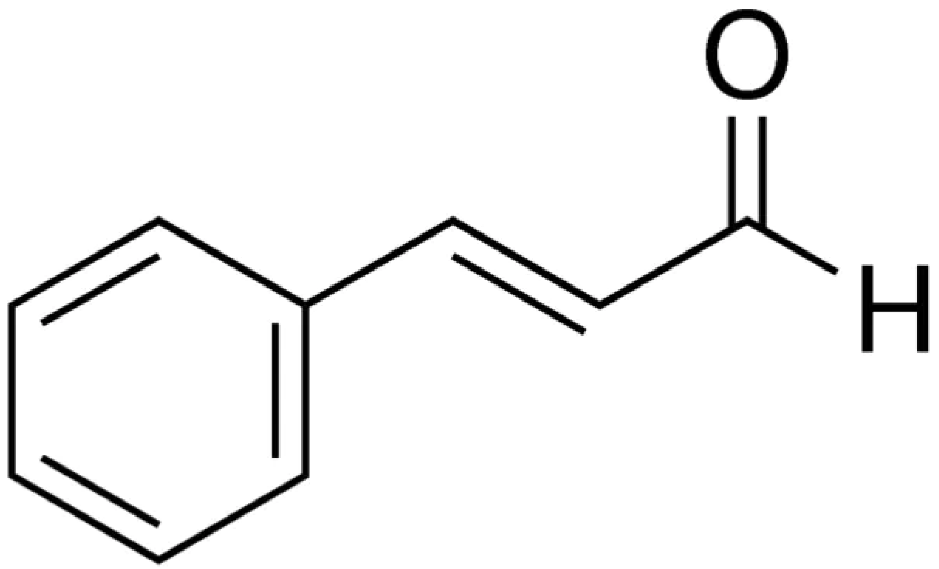

Supplementary Figure S1.   Chemical structure of trans-cinnamaldehyde used in this study.

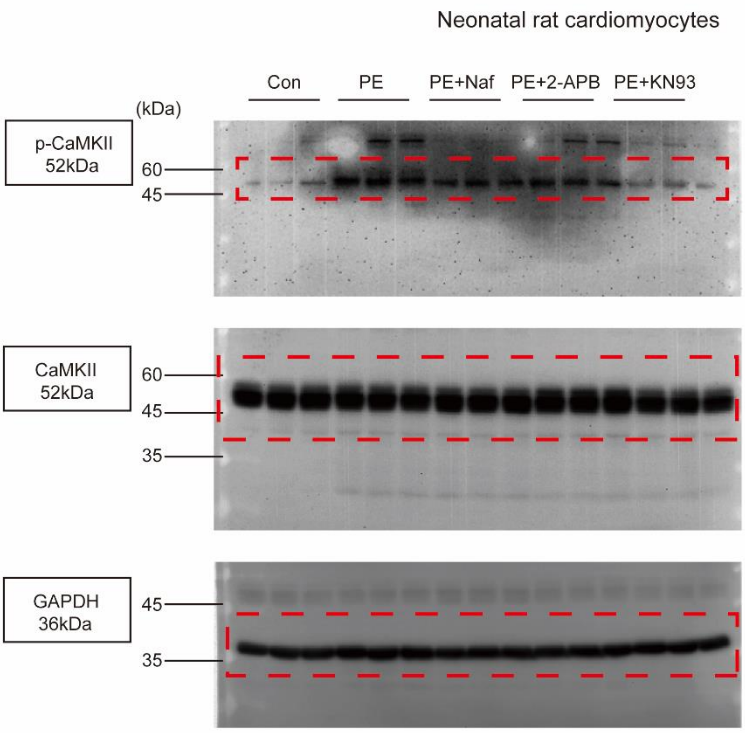

Supplementary Figure S2. The uncropped Western blot images corresponding to Fig.2 A showing all the bands.

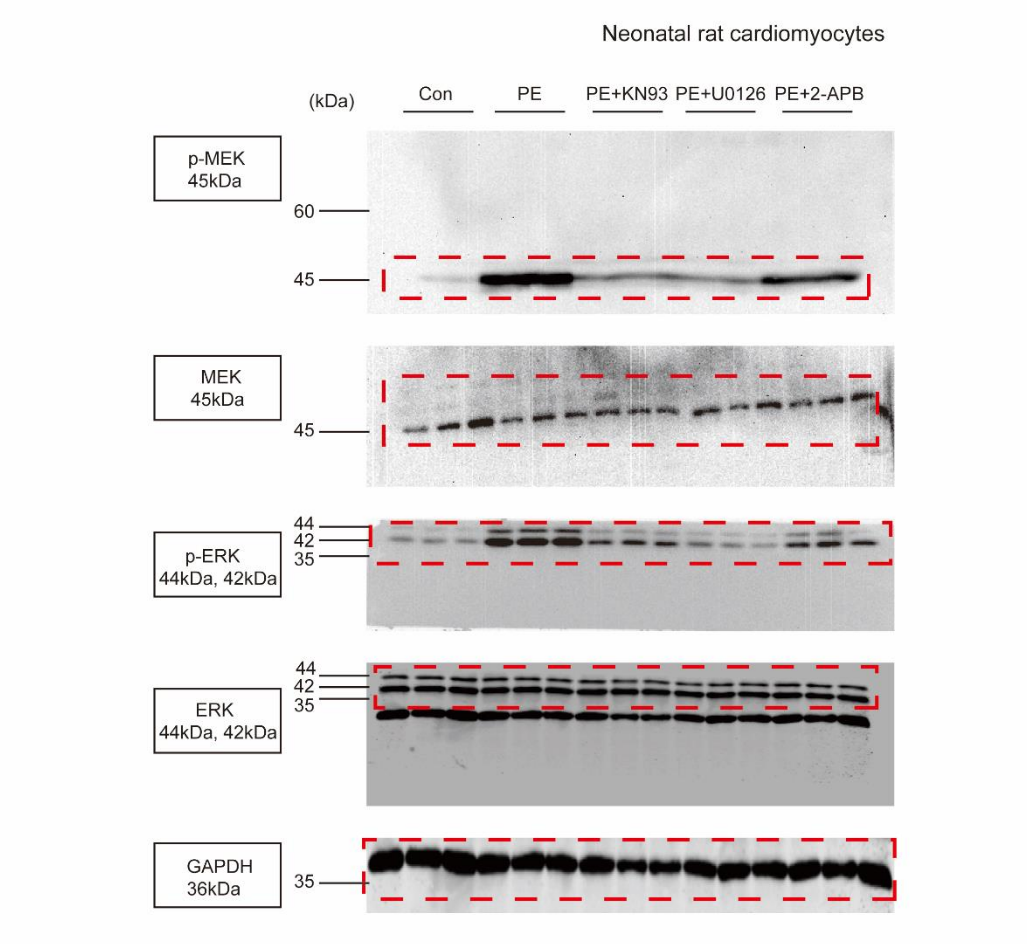

Supplementary Figure S3. The uncropped Western blot images corresponding to Fig.2 C showing all the bands.

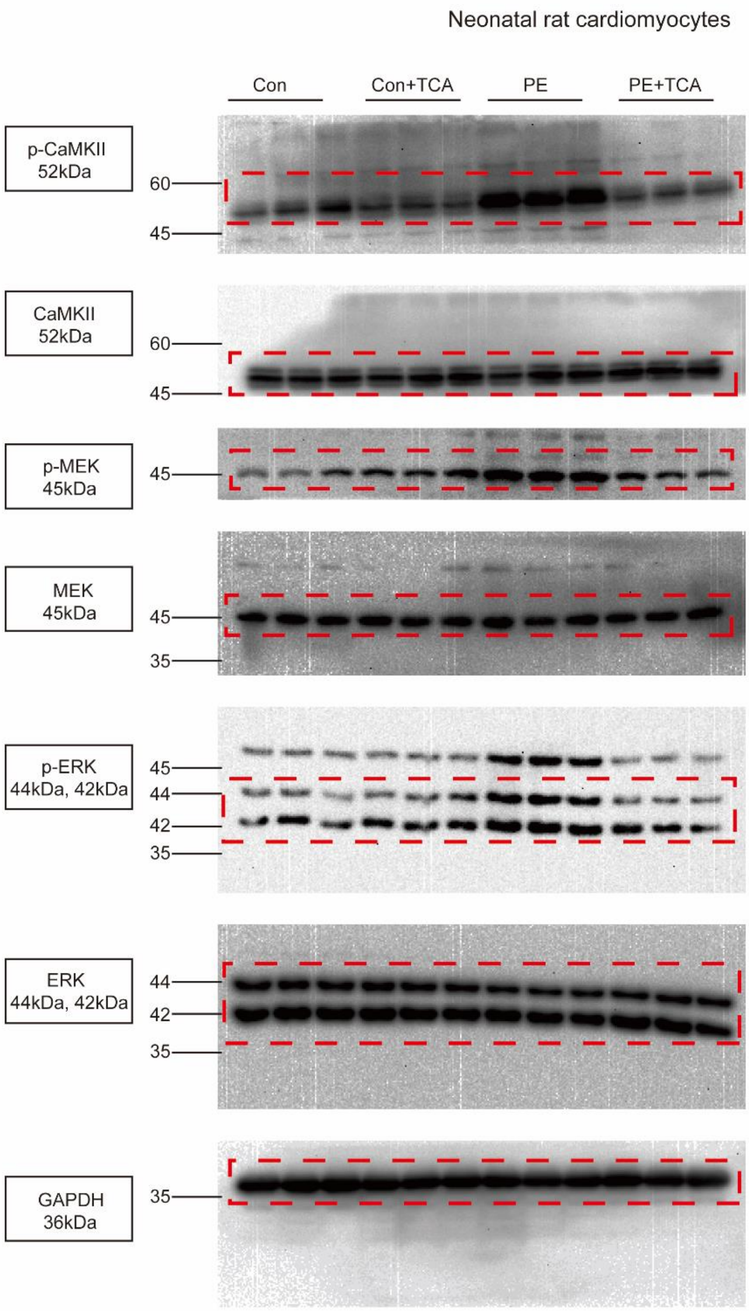

Supplementary Figure S4. The uncropped Western blot images corresponding to Fig.3 A showing all the bands.

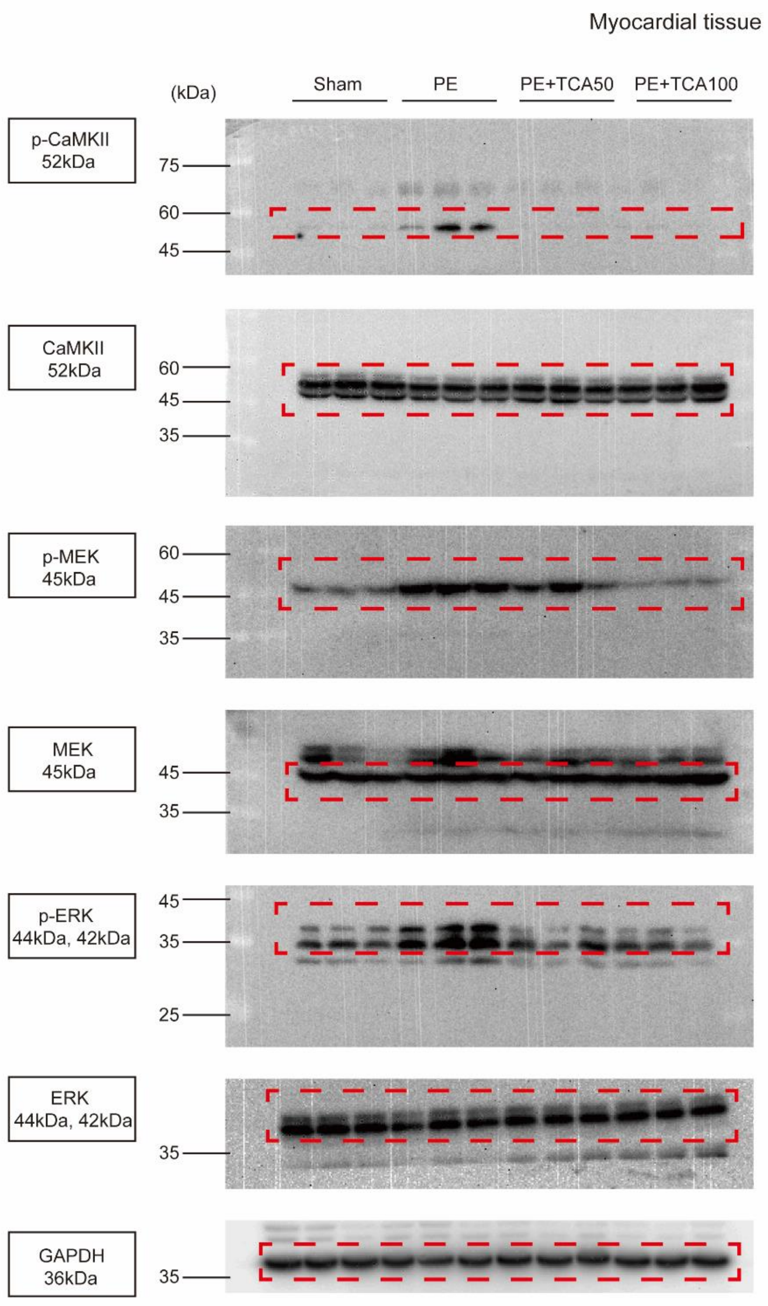

Supplementary Figure S5. The uncropped Western blot images corresponding to Fig.5 F showing all the bands.
